# Temporal and thermal optimization of trypsin digestion for the cryptococcal proteome

**DOI:** 10.1128/mra.01137-24

**Published:** 2025-06-11

**Authors:** Jing Sun, Jason A. McAlister, Jennifer Geddes-McAlister

**Affiliations:** 1 Molecular and Cellular Biology Department, University of Guelph 317113 , Guelph, Ontario, Canada; University of Notre Dame, Notre Dame, Indiana, USA

**Keywords:** proteomics, trypsin, *Cryptococcus neoformans*, workflow optimization

## Abstract

Proteomics investigates diverse biological systems, providing insights into regulation, signaling, modifications, and interactions. Optimized sample preparation is critical to ensure robust and reproducible analyses balanced with time and cost. Herein, we evaluated trypsin digestion parameters for the human fungal pathogen, *Cryptococcus neoformans*, and determined conditions for optimal protein identification.

## ANNOUNCEMENT

In bottom-up, discovery-based proteomics, trypsin is used for sequence-specific protein cleavage for peptide generation (1). Cleavage occurs at the carboxyl end of lysine and arginine residues, producing peptides of a desired length for detection (2). For protein digestion of diverse microbial systems, we traditionally use a 1:50 ratio of enzyme (i.e., trypsin/lysC) to protein with overnight digestion at room temperature or 37°C (3, 4); however, recent studies have suggested optimization of time and temperature can increase digestion efficiency (5).

For the human fungal pathogen, *Cryptococcus neoformans*, designated as a critical-priority pathogen by the World Health Organization (6, 7), identification of proteins associated with fungal virulence and driving host immune response represents targets to combat the pathogen (8–11). For *C. neoformans*, the presence of a thick polysaccharide capsule surrounding the cells presents a barrier to efficient protein extraction (3). Herein, we evaluated the effect of altered time and temperature conditions for trypsin digestion of proteins derived from *C. neoformans* to evaluate proteomic coverage. *C. neoformans* wild-type H99 strain (ATCC 208821) was cultured on yeast peptone dextrose (YPD) agar plates at 37°C overnight. A single colony was transferred to YPD broth (5 mL) and incubated at 37°C with shaking at 200 rpm overnight, followed by a 1:100 dilution into fresh YPD broth for growth to mid-log phase (approx. 8 h). For sample collection, 1 mL of liquid culture was centrifuged at 3,500 rpm, the supernatant was discarded, and the cell pellet was washed twice with 1 mL phosphate-buffered saline (PBS). Samples were prepared for proteomic analysis in biological quadruplicate (3), with modifications to digestion parameters. Briefly, cell pellets were resuspended in cold 100 mM Tris-HCl (pH 8.5) with a protease inhibitor tablet (Roche) and lysed via probe sonication (Fisherbrand Model 505; 30% power, 30 s on/30 s off, ice-bath), followed by the addition of dithiothreitol (10 mM final concentration) at 95°C for 10 min with shaking (800 rpm), cooling to room temperature, and addition of iodoacetamide (5.5 mM final concentration). Samples were subjected to acetone precipitation overnight at −20°C, followed by washing with ice-cold acetone (80%) and resuspension in 8 M urea/40 mM HEPES. Protein concentration was measured by a bovine serum albumin tryptophan assay (12). Protein digestion was performed with trypsin/lysC at a 1:50 ratio (enzyme: protein) at four tested conditions: i) room temperature overnight, ii) 37°C overnight, iii) 37°C for 1 h, and iv) 47°C with 10 mM CaCl_2_ for 1 h (5). Temperatures and times for options 1–3 were selected based on standard proteomics workflows for *C. neoformans* (3). Following the addition of stopping solution (20% acetonitrile; 6% trifluoroacetic acid) at a ratio of 1:10 (vol/vol), the resulting peptides were purified using STop And Go Extraction (STAGE) tips (13).

The samples were measured on an Orbitrap Exploris 240 hybrid quadrupole-orbitrap mass spectrometer (Thermo Fisher Scientific) coupled to a Vanquish Neo liquid chromatography system (Thermo Fisher Scientific). Samples were loaded onto an in-line 75 µm by 50 cm PepMap Spray column filled with 2 µm C_18_ reverse-phase silica beads (Thermo Fisher Scientific). A linear gradient of 3% to 45% buffer B (80% acetonitrile; 0.5% acetic acid) over 120 min was performed for peptide separation, followed by a 100% buffer B wash at a flow rate of 250 nL/min. Full scans (400 to 2,000 *m*/*z*) were acquired with a resolution of 120,000 at 200 *m*/*z*. Analysis of raw mass spectrometry files was performed with MaxQuant (v1.6.0.26) (14) using the Andromeda search engine (15) against *C. neoformans* var. *grubii* serotype A (strain H99/ATCC 208821) FASTA (7,429 sequences; 10 July 2024) from UniProt (16). Trypsin enzyme specificity was set to a maximum of two missed cleavages and a minimum peptide length of seven amino acids, with fixed modifications of carbamidomethylation of cysteine and variable modifications of methionine oxidation and N-acetylation. A false discovery rate (FDR) of 1% with a minimum of two peptides required for protein identification was set for peptide spectral match. Data were analyzed and visualized using Perseus (v2.0.11) (17) and ProteoPlotter (18). Valid value filtering for identification in three of the four replicates within at least one group and imputation based on the normal distribution was performed.

**Fig 1:**
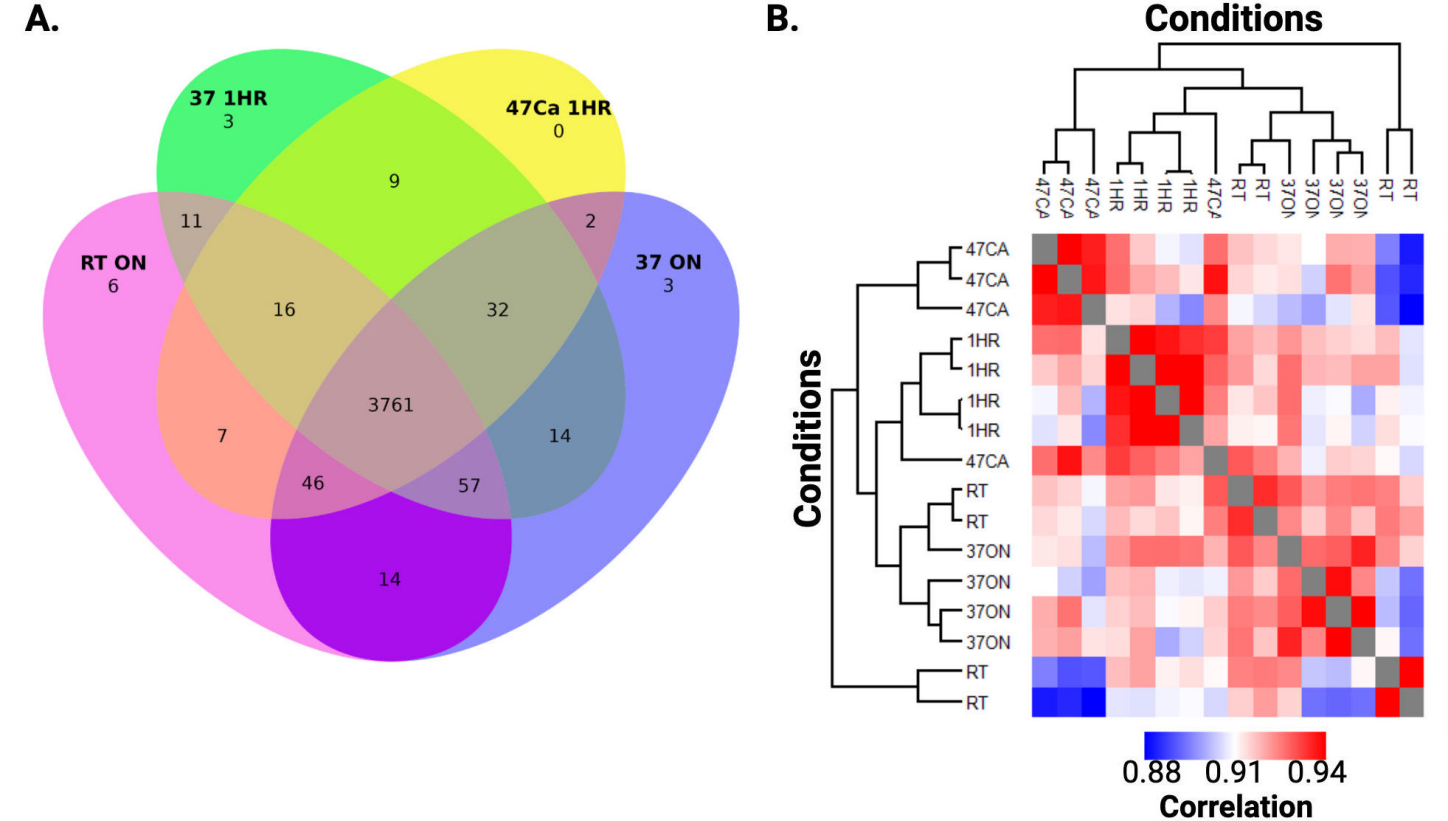
Cryptococcal protein identification rates and replicate reproducibility for optimization of trypsin digestion time and temperature. (**A**) Number of proteins identified across the conditions. (**B**) Hierarchical clustering by Euclidean distance for biological replicates. Correlation values across four biological replicates used to calculate the replicate reproducibility. RT (ON) = room temperature overnight; 37 ON = 37°C overnight; 1 HR or 37 1HR = 37°C for 1 h; 47 Ca or 47 Ca 1HR = 47°C with 10 mM CaCl_2_ for 1 h.

Across the digestion conditions, we observed a core proteome of 3,761 proteins (proteomic coverage of 50.6%) (Fig. 1A), supporting the consensus of peptide generation and protein identifications among the different samples. Additionally, we observed proteins uniquely identified at room temperature overnight (six proteins), 37°C overnight (three proteins), and 37°C for 1 h (three proteins); no unique proteins were identified at 47°C for 1 h. Notably, reducing the digestion step from overnight to 1 h did not appear to negatively impact protein identification rates. We also assessed replicate reproducibility by hierarchical clustering by Euclidean distance and reported a strong correlation across replicates: 92.7% (room temperature overnight), 93.3% (37°C overnight), 94.2% (37°C for 1 h), and 93.5% (47°C with 10 mM CalCl_2_ for 1 h) (Fig. 1B). Overall, this study supported flexibility within our current trypsin digestion protocol to reduce the amount of time required for sample processing, when logistically advantageous, without compromising in protein identification rates and replicate reproducibility.

## Data Availability

The mass spectrometry-based proteomics data are available through PRIDE Proteome Xchange with accession number: PXD056755.
